# Assessment of recruitment from CT to the bedside: challenges and future directions

**DOI:** 10.1186/s13054-025-05263-4

**Published:** 2025-02-06

**Authors:** Stefano Giovanazzi, Domenico Nocera, Giulia Catozzi, Francesca Collino, Massimo Cressoni, Lorenzo Ball, Onnen Moerer, Michael Quintel, Luigi Camporota, Luciano Gattinoni

**Affiliations:** 1 Department of Anesthesiology, University Medical Center Göttingen, Robert-Koch-Str 40, 37075 Göttingen, Germany; 2 Department of Medical and Surgical Specialties, Radiological Sciences and Public Health, University of Brescia, Piazzale Spedali Civili 1, 25121 Brescia, Italy; 3 Department of Medical and Surgical Sciences, Alma Mater Studiorum, University of Bologna, Via Massarenti 9, 40138 Bologna, Italy; 4 Department of Health Sciences, University of Milan, Via Festa del Perdono 7, 20122 Milano, Italy; 5 Department of Anesthesia, Intensive Care and Emergency, AOU Città Della Salute E Della Scienza Di Torino, Corso Bramante 88, 10126 Turin, Italy; 6 Unit of Radiology, IRCCS Policlinico San Donato, Via Morandi 30, 20097 San Donato Milanese, Italy; 7 Department of Surgical Sciences and Integrated Diagnostics, University of Genoa, Genoa, Italy; 8 Anesthesia and Intensive Care, San Martino Policlinico Hospital, Genoa, Italy; 9 Centre for Human & Applied Physiological Sciences, School of Basic & Medical Biosciences, King’s College London, London, UK; 10 Guy’s & St Thomas’ NHS Foundation Trust, London, UK

**Keywords:** Acute respiratory distress syndrome, Recruitment, Mechanical ventilation, Positive end expiratory pressure

## Abstract

Assessing and quantifying recruitability are important for characterizing ARDS severity and for reducing or preventing the atelectrauma caused by the cyclic opening and closing of pulmonary units. Over the years, several methods for recruitment assessment have been developed, grouped into three main approaches: 1) Quantitative CT Scanning: This method accurately measures the amount of atelectatic lung tissue that regains aeration; 2) Regional Gas Volume Measurement: Based on anatomical markers, this approach assesses gas volume within a specified lung region; 3) Compliance-Based Gas Volume Measurement: This technique compares actual gas volume at a given pressure to expected values, assuming respiratory system compliance is constant within the explored pressure range. Additional methods, such as lung ultrasonography and electrical impedance variation, have also been explored. This paper details the distribution of opening and closing pressures throughout the lung parenchyma, which underpin the concept of recruitability. The distribution of recruitable regions corresponds to atelectasis distribution, with the pressure needed for recruitment varying according to whether the atelectasis is “loose” or “sticky.” We also discuss the effects of different PEEP levels on preventing atelectrauma, the importance of keeping some lung areas closed throughout the respiratory cycle, and briefly cover the roles of sigh ventilation, prone positioning, and the closed lung approach.

## Background

The term *recruitment*, originally a military term, denotes a shift from one status (civilian) to another (soldier). In lung physiology, however, it represents a type of transition, and in the acute respiratory distress syndrome (ARDS) literature, *recruitment* is used to describe three distinct processes:

1. Reaeration of previously collapsed (degassed) pulmonary units—typically assessed through quantitative computed tomography (CT) scans;
2. Increase in gas content within a defined lung region—identified using morphologic CT scan and Lung Ultrasound Sonography (LUS);
3. The sum of the reaeration of previously gasless pulmonary units in addition to greater inflation of already open units—measured via exhaled gas concentration methods or Electrical Impedance Tomography (EIT).

While often incorrectly considered interchangeable, each method to assess recruitment will yield vastly different results and quantification of recruitability. In this review, we aim to summarize the different methods used for assessing recruitment, their clinical relevance, and their impact on lung physiology during mechanical ventilation in ARDS patients. In Table 1, we summarize the recruitment assessment methods, highlighting their original description.

**Table 1:** Methods to assess recruitability through years

Method	Year	Author	Mechanism
Computed tomography (CT)	1987	Gattinoni et al.[16]	Morphology, Quantitative analysis
Gas methods	1984 1991 1998 1999 2020	Matamis et al.[49] Ranieri et al.[19] Gattinoni et al.[20] Jonson et al. [21] Lu Chen et al. [22]	P/V curve analysis: differences between expected and measured gas volumes when changing pressure
Lung ultrasound sonography (LUS)	2008 2019	Bouhemad et al. [28] Chiumello et al. [30]	Based on change of gas/tissue ratio
Electrical impedance tomography (EIT)	2007 2009 2019	Meier et al.[25] Costa et al. [24] Jonkman et al. [23]	Based on changes of impedance

## Recruitment: opening and closing pressure

**Fig. 1:**
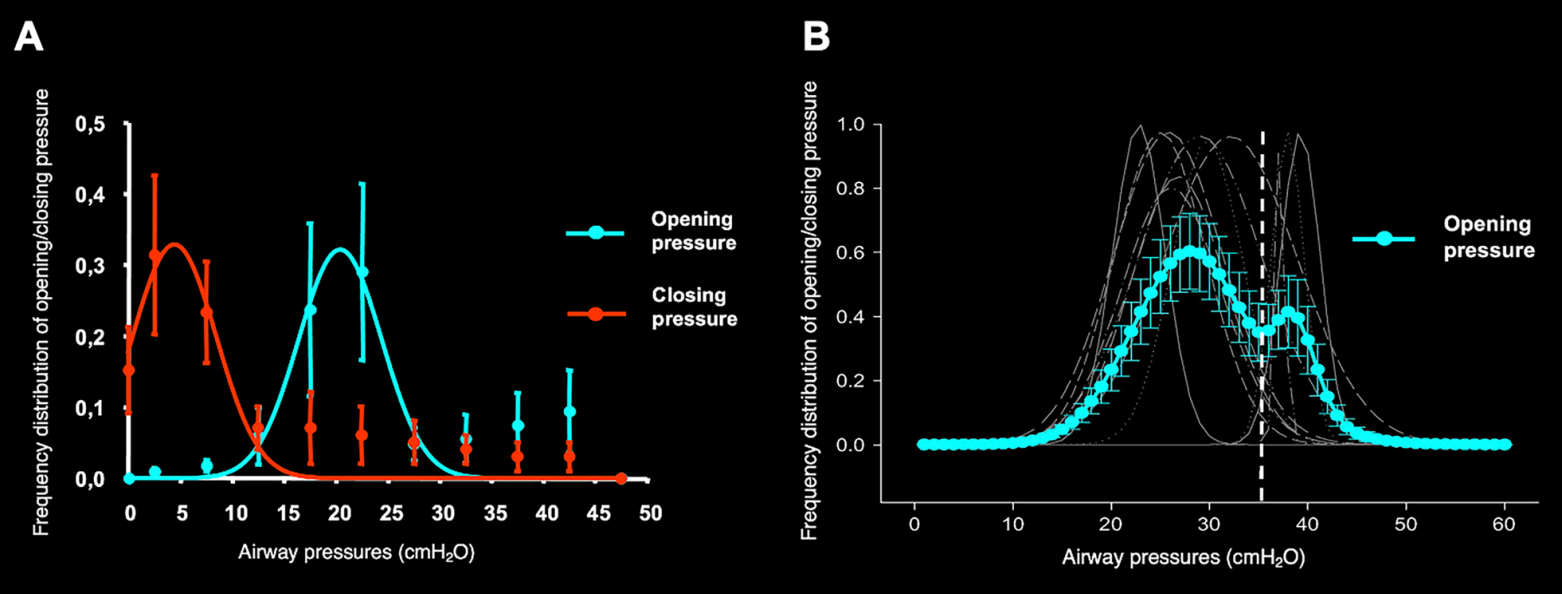
Opening and closing pressures. Frequency distribution of estimated threshold opening (blue) and closing pressures (red) as a function of airway pressure. Panel **A** The fitted recruitment (blue) and derecruitment (red) pressure curves, obtained in each patient, computed at 5 cmH_2_O pressure interval. Rearranged from Crotti et al. [1]. Panel **B** The blue line represents the average distribution of opening pressures across patients. Rearranged from Borges et al. [2]

Before discussing ‘recruitability’ and ‘recruitment’, it is essential to define the basic mechanisms underlying recruitment which involve *opening* and *closing* pressures. In Fig. 1, we present two examples illustrating these phenomena. As shown, opening pressures are distributed throughout a range of pressures, with mode values of pressures around 20–25 cmH₂O. At 40–45 cmH₂O, most of the potentially recruitable regions are open, with only an additional 2–3% of residual closed units may still be recruited with pressures between 45 and 65 cmH₂O [1, 2].

**Fig. 2:**
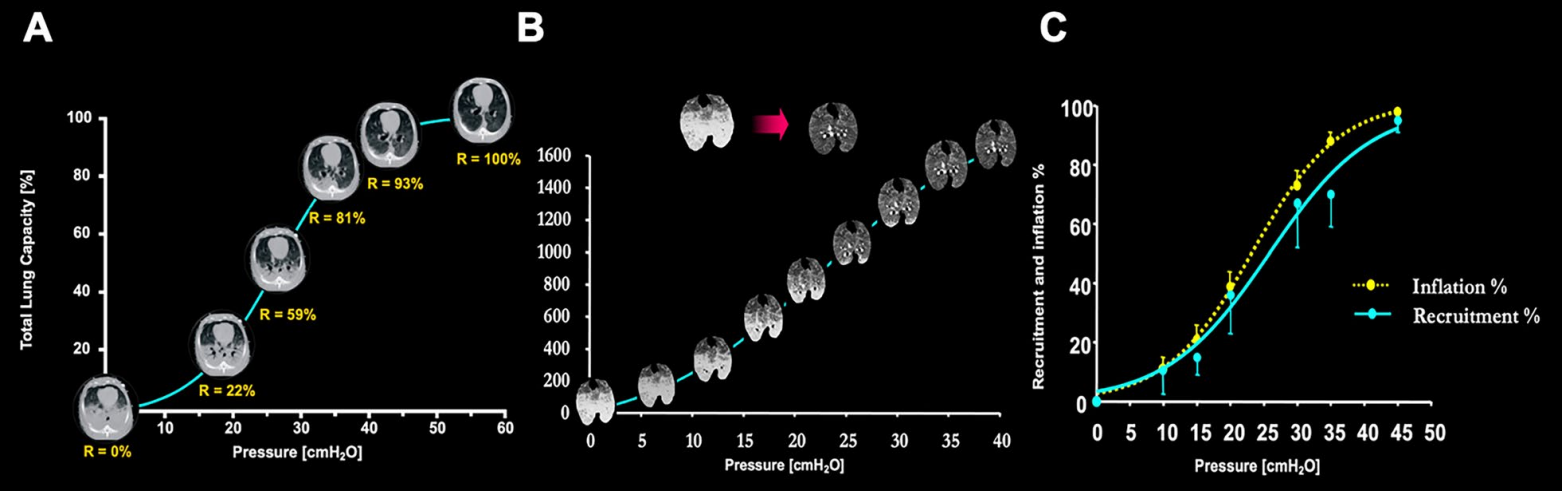
Recruitment in different ARDS models as a function of the applied airway pressures. Panel **A** Experimental ARDS model obtained with oleic acid in dogs. Recruitment occurs along the entire volume/pressure curve, even after the upper inflection point. “R” indicates the percentage of recruitment occurring at the corresponding airway pressure. Rearranged from Pelosi et al. [8]. Panel **B** Experimental ARDS model obtained with oleic acid in pigs. A similar behavior concerning recruitment, as in Panel (**A**). Rearranged from Quintel et al. [9]. Panel (**C**) Percentage of recruitment as a function of airway pressure, which fit a sigmoid function. Rearranged from Crotti et al. [1]

**Fig. 3:**
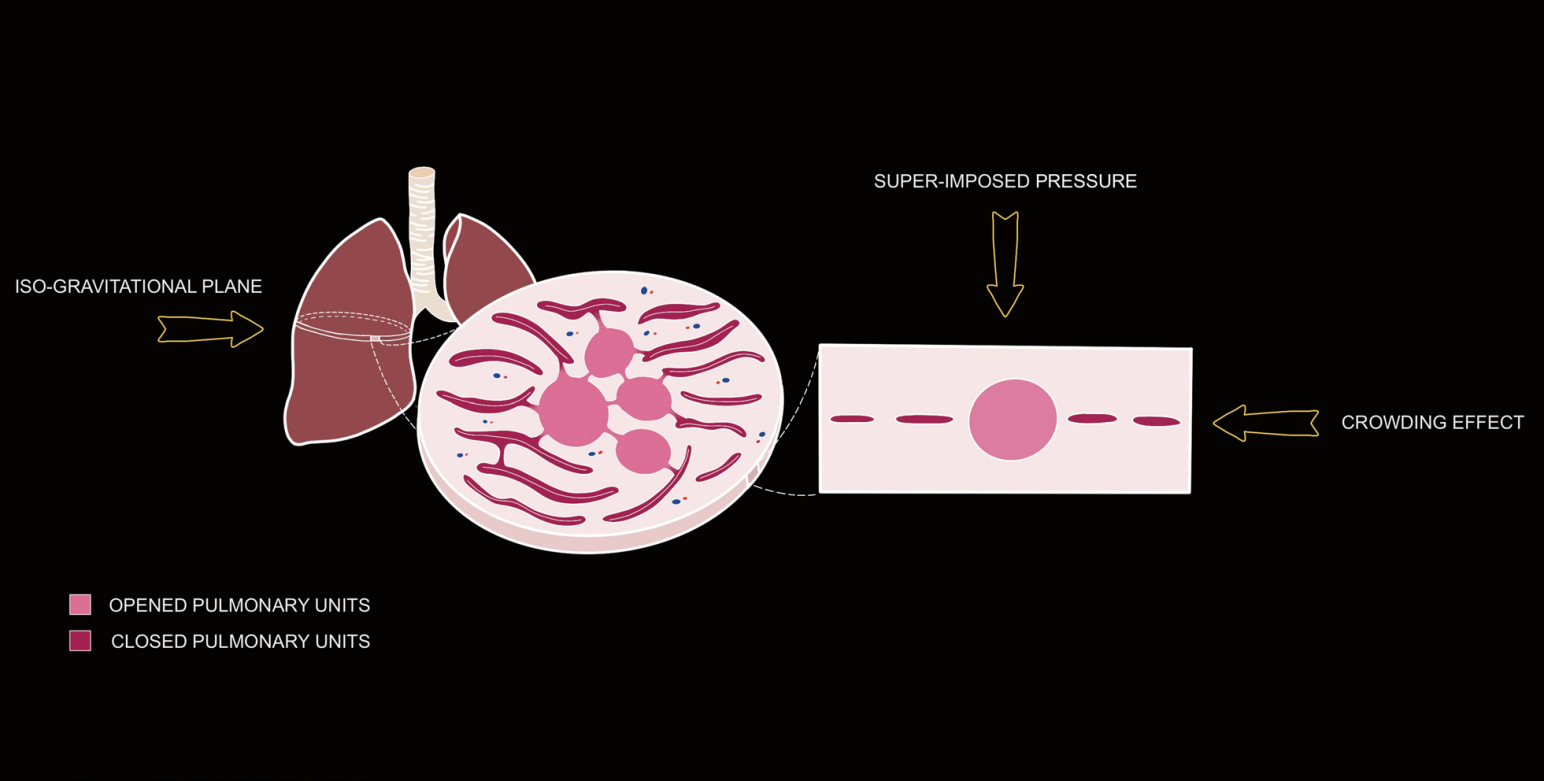
“Crowding effect”. This is a representation of a lung slice, in which the pulmonary units are on the same iso-gravitational plane. At this level some units are open, while others are closed. All of these are subject to the same superimposed pressure. To open a pulmonary unit at this level, we must consider also the “crowding effect”, which is represented by the interaction between neighboring units collapsed. This leads to an increase in opening pressures on an iso-gravitational plane due to the crowding of closed units

**Opening pressure.** Several factors contribute to determining opening pressure. To reopen a collapsed lung, the applied pressure must: 1) overcome the alveolar surface tension (~ 15–20 cmH₂O)[3]; 2) the pressure needed to displace the chest wall (~ 5–10 cmH₂O) [4]; 3) overcome the compressive gravitational forces acting on a given pulmonary unit, which may be ~ 10–15 cmH₂O in the most dependent lung region [5, 6]. Therefore, the total opening pressure for the respiratory system may be estimated in the range of 30–45 cmH₂O [7]. It is worth noting that, at these pressures, the pulmonary units are in the range of their total capacity (i.e., the physical limit of lung expansion). A consequence of the heterogeneous gas distribution and alveolar opening pressures within the lungs, is that the recruitment, both in experimental animals [8, 9] and in humans [1], occurs along the entire pressure/volume (P/V) curve (Fig. 2). This is in contrast with the traditional view which confines the recruitment at the first part of the P/V curve, before the upper ‘inflection point’, and assumes minimal recruitment after this pressure values. The fact that a few pulmonary units open at pressures higher than 60 cmH₂O is likely due to the "crowding effect" representing the alveolar-interdependence of neighboring pulmonary units situated within an iso-gravitational plane (i.e., the additional pressure required to inflate pulmonary units on the iso-gravitational plane), see Fig. 3.

**Closing pressures.** After alveolar opening, which is a function of inspiratory pressure, the lungs deflate during expiration. During this phase some of the pulmonary units may collapse at different pressure according to their own closing pressures. As shown in Fig. 1, closing pressures are substantially lower than the opening ones [1] since the pressure required to overcome the surface forces is not needed during the deflation. This explains why closing pressures are approximately 10–15 cmH_2_O lower than opening pressures.

In summary, recruitment is an inspiratory phenomenon that depends on the opening pressure, while maintenance of recruitment (as in the application of positive end-expiratory pressure [PEEP]) is an expiratory phenomenon that requires a pressure greater than the closing pressure. The effect of PEEP in maintaining recruitment is closely linked to the intrinsic properties of the alveolar units, particularly their closing pressures. While PEEP levels are not directly determined by the potential for lung recruitment, this potential becomes clinically relevant in patients with high closing pressures. In such cases, higher PEEP may be justified due to the potential gain in aeration. Conversely, in patients with low recruitment potential, the same PEEP level may provide minimal aeration benefits while causing overinflation and imposing greater extrapulmonary effects, making its use less beneficial. In other words, although the potential for recruitment does not necessarily imply a need for higher PEEP, the potential gain in aeration may justify its use in individual patients.

## Recruitability (atelectasis) distribution

**Fig. 4:**
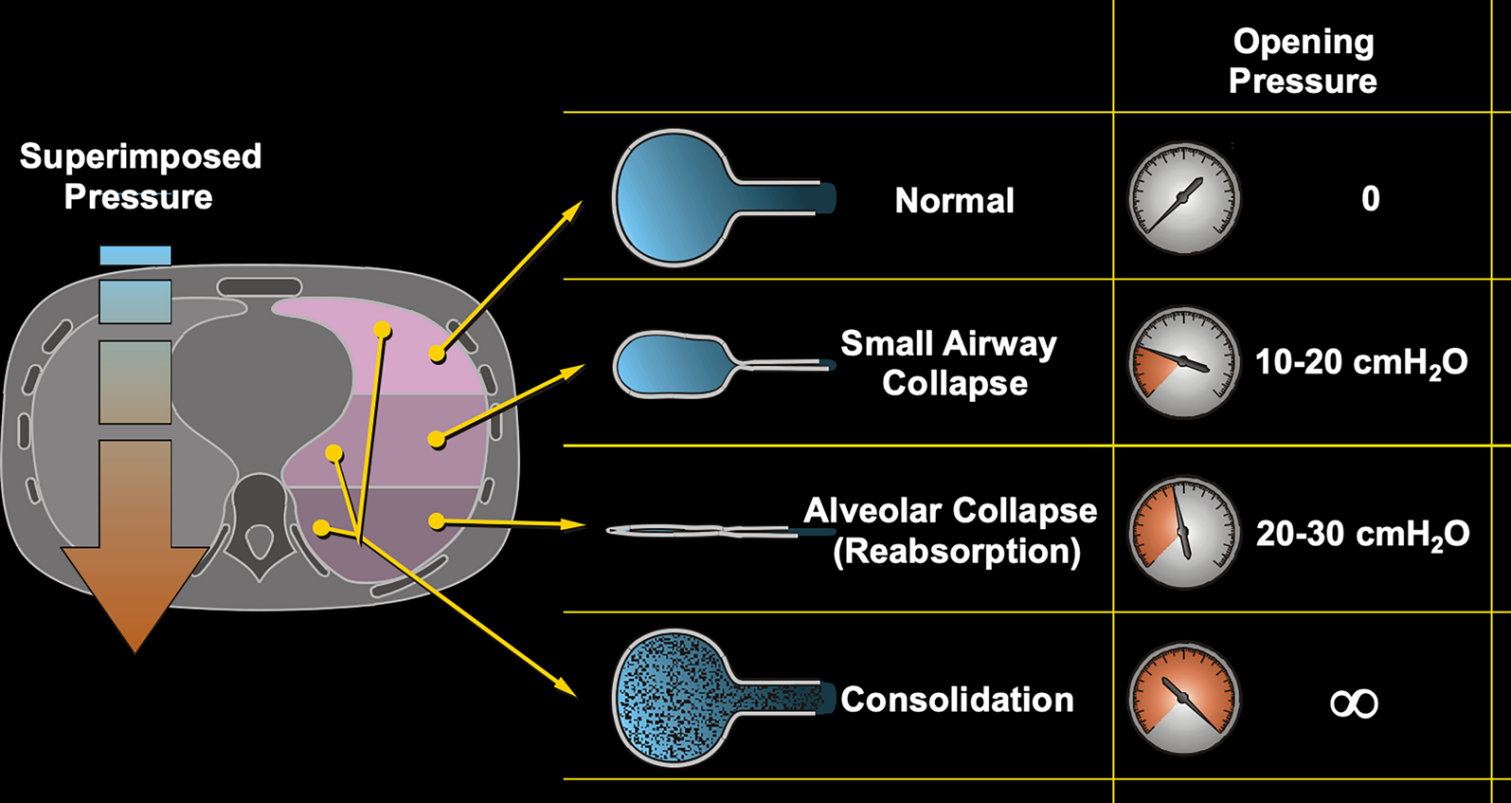
Pulmonary units and their different opening pressures. This schema represents the different type of pulmonary units, their principle spatial orientation and the corresponding opening pressures, according to the superimposed pressure. The “normal aerated” units are already open and have no need for opening pressure. The “loose atelectasis” needs an opening pressure around 10–20 cmH_2_O. The “sticky atelectasis” needs an opening pressure around 20–30 cmH_2_O. The “consolidated” units will not open whatever the pressure is

To model the ARDS lung, it is helpful to view it as a mixture of pulmonary units with different physical status and characteristics (Fig. 4).

- Some lung units, which are the core of the **“baby lung”** concept [10], have a nearly normal inflation status and are primarily located in the non-dependent regions of the lung (ventral in the supine position and dorsal in the prone position). The opening pressure of these units may be considered close to 0 cmH₂O, as they remain open at atmospheric pressure.
- ***Compressive (“loose”) atelectasis.*** These are caused by small airway collapse, which may leave some residual gas into the pulmonary units [11]. These atelectasis are primarily caused by an increase in hydrostatic pressure due to the increase in lung weight. The affected lung units follow a spatial orientation which follows the gravitational axis from the non-dependent to the dependent lung. Across an iso-gravitational plane, not all the lung units have the same inflation status (all open or all closed), but the gas content varies due to the interaction between contiguous units (alveolar interdependence). The unit collapsing at end-expiration remains *“loose”* if they reopen during the next inspiration, being the opening pressure around 10–20 cmH_2_O, otherwise the complete obstruction and reabsorption of gas will require higher pressures to reopen.
- ***Obstructive (resorptive, “sticky”) atelectasis***. These are due to the complete reabsorption of gas from the pulmonary unit, which occurs when the airway remains closed for the entire respiratory cycle [11]. This atelectasis are mostly distributed in the dependent regions, where the compressive forces are higher. Reabsorption atelectasis develops over time when regional gas uptake exceeds delivery, which depends on the F_I_O_2_, regional ventilation/perfusion ratio, and likely the end-expiratory volume of the pulmonary units. Once no gas remains in these units, the transmural pressure required for reopening can reach 30–35 cm H_2_O, corresponding to a plateau pressure exceeding 60 cm H_2_O. It must be noted that the number of collapsed units is a determinant of the increase of the lung weight [12] (i.e., the increase of the superimposed pressure) [5].
- The disease leading to ARDS may also cause a proportion of pulmonary units to be ***“consolidated”.*** In this model *consolidation* refers to pulmonary units which are not only gasless, but their volume is occupied by liquid or solid material (e.g., as intra-alveolar edema, blood, cellular debris). In these units, the opening pressure is close to infinity, as no pressure will be able to increase the gas content. The distribution of consolidated units depends on the nature of the disease, and it is not predictable.

**Fig. 5:**
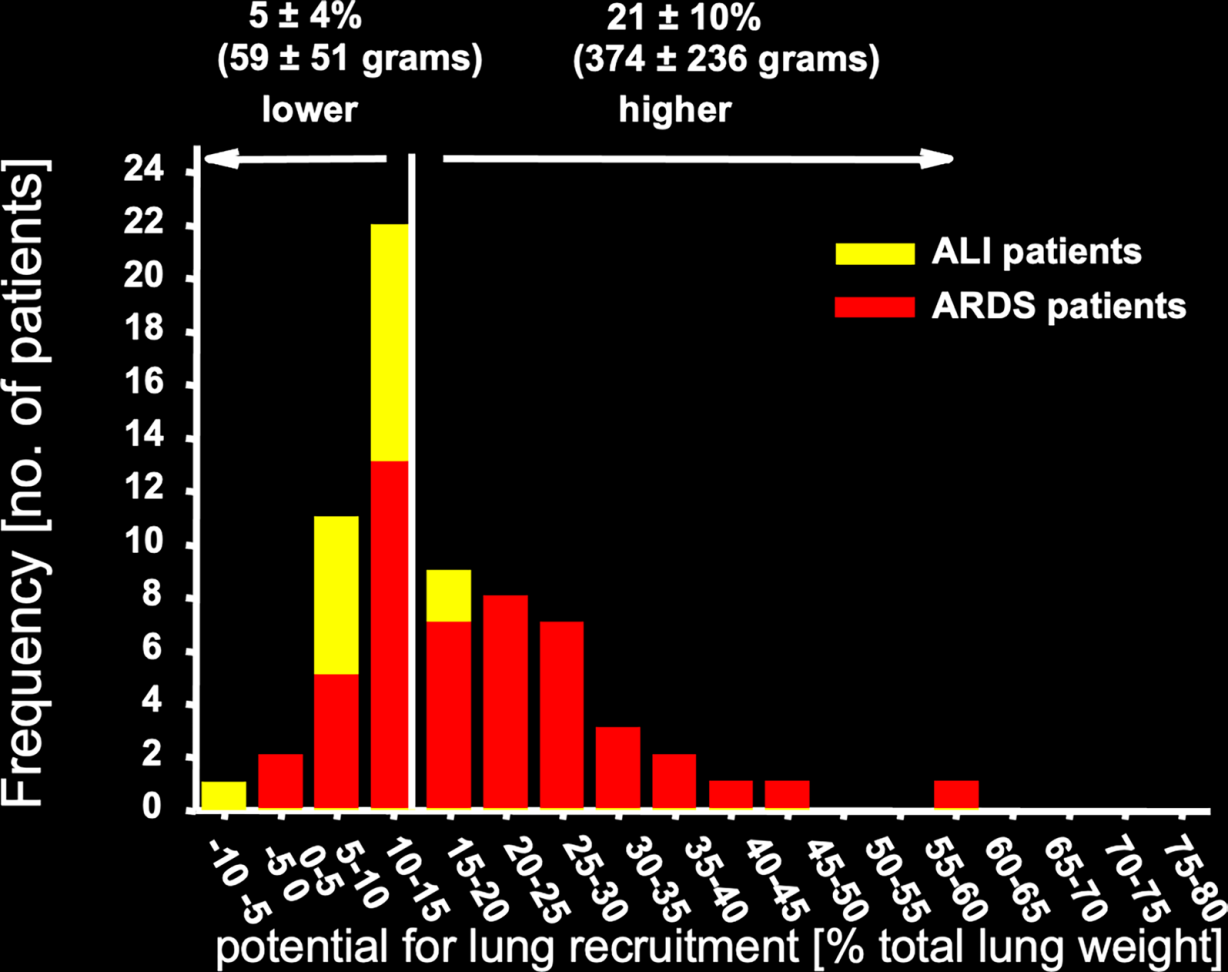
Frequency distribution of patients depending on recruitment. Frequency distribution of patients according to the percentage of potentially recruitable lung, expressed as the percentage of total lung weight. The percentage of potentially recruitable lung was defined as the proportion of lung tissue in which aeration is restored at airway pressures between 5 and 45 cmH_2_O. Rearranged from Gattinoni et al. [12]

This model accounts for two phenomena. First, recruitability (i.e., the proportion of potentially openable units) is proportional to the lung weight and therefore the increased superimposed pressure. Second, it accounts for the significant variability observed in patients when recruitability is measured, as shown in Fig. 5 [12].

## Recruitment assessment by computed tomography

### Morphology

**Fig. 6:**
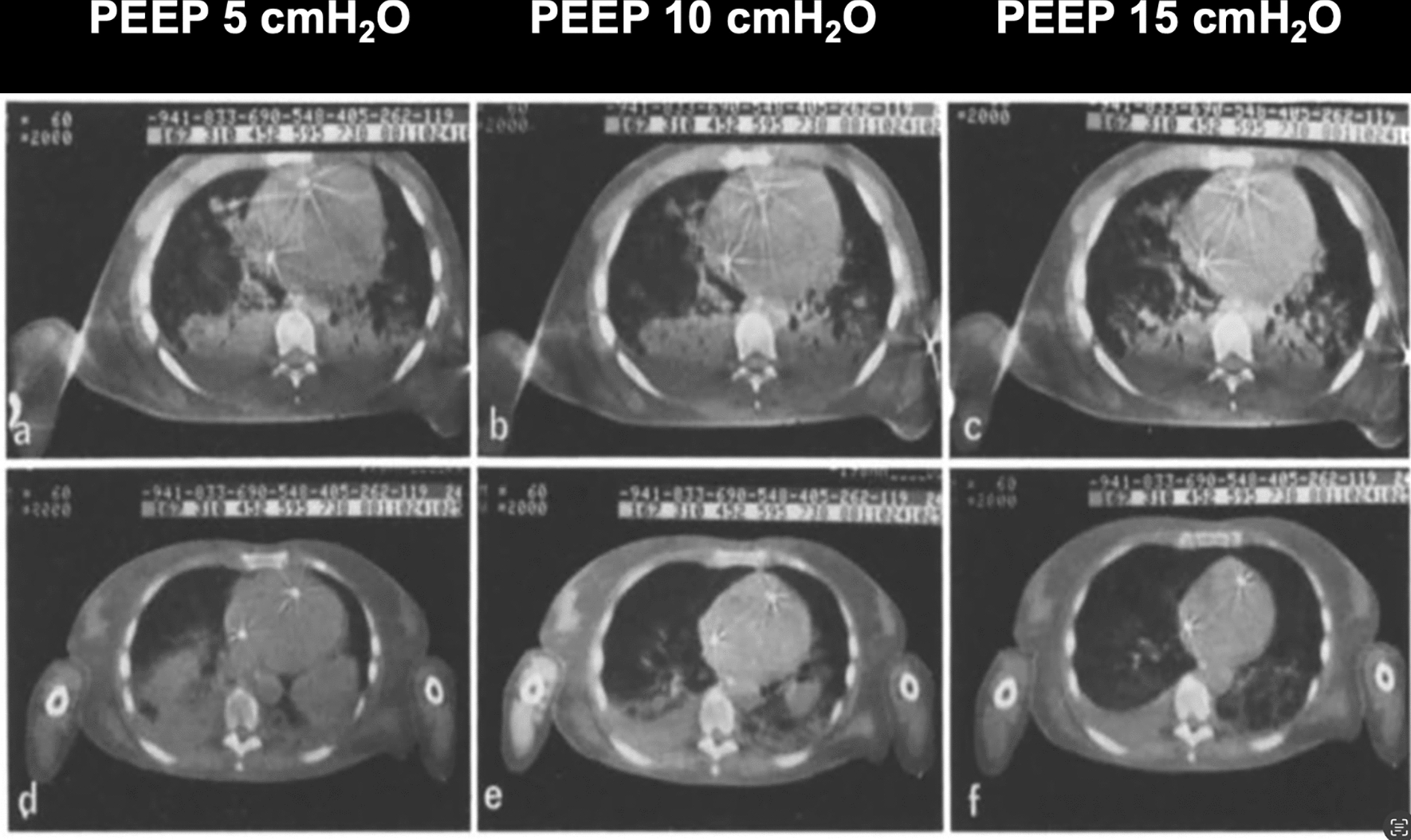
CT scan of the base at 5, 10 and 15 cmH_2_O. Upper line **a**–**c** Acute respiratory failure caused by bacterial pneumonia. The three different CT scans at varying level of PEEP show no substantial differences, no recruitment leading to the risk of overdistention. Lower line **d**–**f** Acute respiratory failure cause by sepsis. The densities are markedly reduced with the increase of the PEEP, meaning higher recruitment. Rearranged by Gattinoni et al. [13]

In 1986, we were the first to report the effect of PEEP in maintaining the lung at least partially open at end-expiration, preserving recruitment achieved during the preceding inspiration [13]. In Fig. 6, we present the first image demonstrating the measurement of the open lung based on density dimensions assessed via CT scan. This image highlights the impact of PEEP on lung densities (i.e., recruitment) and its hemodynamic effects. It compares a case where the recruitment was minimal and another where significant alveolar recruitment occurred. The degree of lung volume change affected the cardiac silhouette, which remained largely unaltered when the increase the increase in airway pressure did not result in an increase in lung volume. However, when lung volume increased either as a consequence of true alveolar recruitment (opening of gasless alveolar units) or inflation (increase in gas content of already opened units), the cardiac volume decreased as a consequence of an increase in pleural and pericardial pressures. This figure illustrates also the interplay between hemodynamics and oxygenation, as changes in oxygenation could not be explained by changes in the density of the lung parenchyma alone indicating the changes in the distribution of pulmonary blood flow is an important contributor to gas exchange.

The morphological analysis of recruitability was strongly promoted by Rouby’s research group [14] and more recently applied clinically by Constantin et al. [15] in guiding PEEP settings.

### CT quantitative analysis

**Fig. 7:**
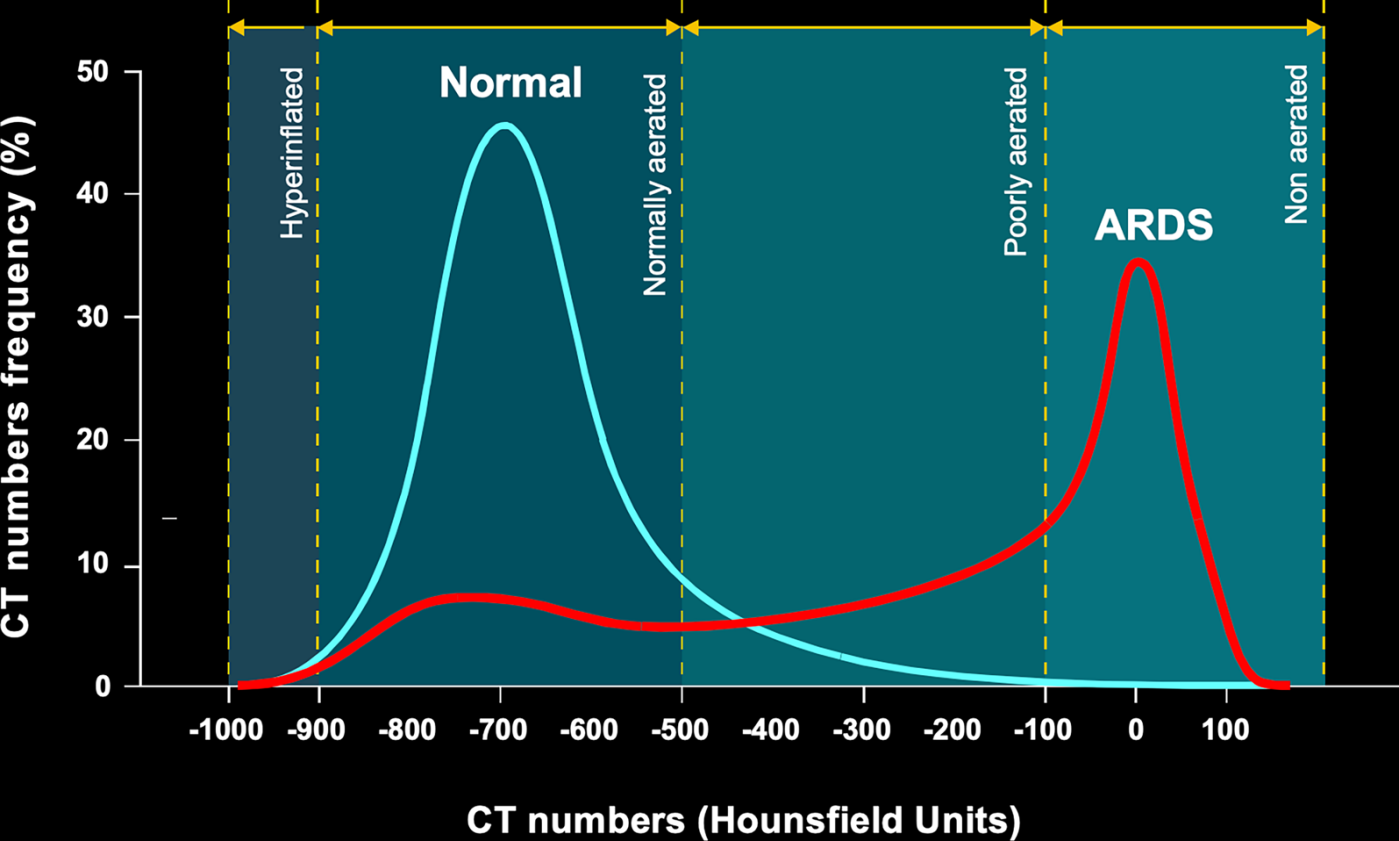
CT frequency distribution as a function of CT numbers, compared between normal subjects and ARDS patients. Four different zones, defined according to the CT scan quantitative analysis thresholds, are shown. The distribution of patients with acute respiratory failure (red line) is primarily in the poorly aerated and non-aerated zones, whereas the distribution of normal subjects (blue line) shows a predominance in the normally aerated zone. Rearranged from Gattinoni et al. [16]

The quantitative analysis, using CT Hounsfield Units (HU) thresholds to classify lung regions as *non-aerated* (radiodensity between + 100 and − 100 HU), *poorly aerated* (radiodensity between − 101 and − 500 HU), *normally aerated* (radiodensity between − 501 and − 900 HU), and *hyperinflated* (radiodensity between –901 and –1000 HU), was introduced in 1987 [16]. This approach based on the analysis of HU allows estimating the lung density, assuming that air (-1000 HU) has a density of 0 g/ml, while deflated lung tissue has a density equal to that of water, namely 1 g/ml. A specific HU number in a lung region reflects its density, determined by the ratio of air to water content in that area [17]. This classification based on HU thresholds has since been widely adopted and requires an analysis of the distribution of CT values, as shown in Fig. 7. A complete report for the CT scan thresholds used in the literature between 1986 and 2016 is reported by Chiumello et al. [18]. The most significant consequence of this CT-based quantitative analysis was to realize that compliance and elastance reflect the size of the open lung (i.e., the lung is not stiff, but it is small). The strict relationship between gas volume and compliance/elastance is the physiological basis of the recruitability assessment by gases, which are all based on changes of respiratory system compliance.

## Bedside assessment

### Gas methods

The gas methods are based on the comparison of expected changes in volume/pressure when inflating the lung with the volume/pressure actually measured. As an example, for a given respiratory system compliance we expect a certain volume change when applied pressure is increased (as an example from 5 to 15 cmH_2_O), this expected volume is the product of compliance at low pressure and the applied variation of pressure (in this case 10 cmH_2_O). If the volume measured at 15 cmH_2_O is greater than the one expected, the volume difference is attributed to recruitment. All the gas methods rely on two assumptions: first that the compliance of the newly opened units is equal to the compliance of the already opened units; and second that the pressure/volume curve is substantially linear in the range of pressures applied. Here below we summarize the gas methods.

1. The first to use the P/V curves method was Ranieri et al. in 1991 [19], based on the construction of two P/V curves obtained at two different pressure levels, (PEEP and zero end expiratory pressure [ZEEP]). These were obtained performing inflation volumes and plotting them with the corresponding pressures, measured by performing static end-expiratory airway occlusion. The difference of lung volume between PEEP and ZEEP at the same static pressure was assumed to be the gas volume recruited.
2. In 1998 Gattinoni et al. [20] estimated lung recruitment by measuring the lung gas volume at two different levels, PEEP and zero PEEP (ZEEP). The End Expiratory Lung Volume (EELV) at ZEEP (namely the functional residual capacity, FRC) was measured using a closed-circuit helium dilution method. The lung volume at PEEP was computed by measuring the exhaled volume during the transition from PEEP to ZEEP, during an expiratory period long enough to reach an end-expiratory flow of zero. Consequently, EELV was calculated as the sum of the FRC (helium) and the difference between the exhaled volume and the tidal volume. The difference between EELV and FRC was assumed to be the recruited volume. To estimate the lung recruitment by PEEP, these methods differentiate the component caused by the *inflation* of pulmonary units already open and the component caused by the *recruitment* of previously collapsed pulmonary units, calculating the predicted volume adding the EELV at ZEEP, considering the compliance measured at ZEEP.
3. In 1999 Jonson et al. [21] analyzed the P/V curve focusing on the elastic pressure of the system, eliminating the resistive part. Measuring the elastic pressure–volume (Pel–V) curves of the respiratory system and the lung volume recruited by applying PEEP gives some clarification into the pressure range over which recruitment occurs in cases of ARDS. This approach also helps to explain how recruitment influences lung compliance. High compliance during insufflation from ZEEP suggests that lung recruitment occurs well above the lower inflection point on the Pel–V curve. This difference in volume and compliance became negligible at pressures exceeding 30 cmH₂O, indicating that continuous lung recruitment occurred as the pressure was increased from ZEEP up to 30 cmH₂O. Reaching pressures of 30 cmH₂O or higher was necessary to achieve full lung recruitment, a finding consistent with the results reported by Gattinoni et al. [20].
4. In 2020, Lu Chen et al. [22] proposed the recruitment-to-inflation ratio (R/I ratio) which compared the compliance of the recruited volume (C_rec_), obtained with two different levels of PEEP (i.e., at 5 and 15 cmH_2_O), and the compliance of the respiratory system at the lower PEEP. C_rec_ was calculated doing the ratio between the recruited volume (V_rec_) and the difference between PEEP_high_ and PEEP_low_. A de-recruitment manouevre with a single breath, without disconnecting the patient from the ventilator, was performed to calculate the recruited volume; V_rec_ was obtained by the difference between ΔEELV_measured_ and ΔEELV_ideal_. The first one was obtained with a single breath dropping from PEEP_high_ to PEEP_low_ and computing the difference between the exhaled volume after the drop and the expired tidal volume with high PEEP. The second one was calculated as the product of compliance at PEEP_low_ and the difference between PEEP_high_ and PEEP_low_.

The limitations of all these methods are that they cannot differentiate between recruitment and overinflation of the pulmonary units. Therefore, these methods can be used to roughly estimate recruitment, but not to balance the risks of de-recruitment with those of hyperdistention.

### Electrical impedance tomography

Electrical impedance tomography (EIT) is gaining popularity as a bedside, non-invasive, and radiation-free imaging tool for the lungs. This technique employs multiple electrodes placed on the chest to apply a low-voltage current, which allows for the measurement of changes in body impedance. EIT does not produce images of lung aeration like a CT scan but instead offers dynamic real-time visualization of ventilation distribution and changes in lung volume, capturing both global and regional changes in lung volumes based on ventilator settings [23].

By measuring relative changes in voxel compliance, it is possible to estimate the percentage of potentially recruitable alveolar collapse. To calculate EIT-derived regional compliance, changes in pixel impedance during each breath are divided by the airway driving pressures [24]. This can be achieved by monitoring changes during PEEP titration to estimate the percentage of collapsed lung units visible on CT scans and to identify recruitable lung areas. The process requires a reference value obtained at a high PEEP level and involves performing a decremental PEEP trial across a wide range (typically from 24 to 6 cmH₂O). A decrease in voxel compliance when lowering PEEP suggests lung collapse, while a decrease in voxel compliance when increasing PEEP indicates overdistention [25]. The most common clinical application of this method is setting PEEP around the PEEP level where the curves representing the amount of recruitment and overdistension intersect. While physiologically sound, this method implies that overdistension and recruitment are equally harmful, which is probably an oversimplification [26]. Moreover, EIT measures volumes: given the large difference in density between atelectasis and overinflated regions, the change in volumes do not correspond to the amount of lung tissue.

### Ultrasound

For ARDS patients, LUS is helpful for analyzing both overall and specific lung aeration. It helps assess the severity of the illness, guides treatment decisions, and monitors the patient’s response.

In ARDS, specific ultrasound patterns indicate different levels of lung aeration: *Normal aeration*: horizontal A lines; *Moderate decrease in aeration*: multiple vertical B lines (comet tails); *Severe decrease in aeration*: coalescent B lines; *Complete loss of aeration*: lung consolidation with white points and dynamic bronchograms [27, 28].

In 2008 Bouhemad et al. [28], emphasized that bedside ultrasound is effective for assessing lung recruitment induced by PEEP, providing valuable insights into lung function without the need for sedation or complex procedures. However, Chiumello et al. demonstrated a correlation between the percentage of non-aerated, poorly aerated, and normally aerated lung tissue observed in quantitative CT analysis and the LUS score [29]. This suggests that LUS can effectively reflect variations in lung aeration. However, this change in aeration include both *alveolar inflation* (increased aeration of partially inflated units) and *recruitment* (opening of gasless alveoli). Therefore, the global LUS score does not correlate with *alveolar recruitment* measured using CT, which is strictly defined as the reduction of non-aerated tissue [30]. This surprising discrepancy indicates that while LUS is a reliable tool for assessing lung aeration, it may not adequately capture the dynamics of alveolar recruitment under certain conditions, underscoring the need for a comprehensive approach to lung assessment in critically ill patients.

## Differences between recruitment assessment methods and clinical implications

The quantitative assessment of recruitment with the CT scan results in recruitment values, which are markedly different from the ones measured with the gas methods which, in contrast, leads to results similar of CT morphology-based method. Indeed, Chiumello et al. [18] found that changes in airway pressures between 5 and 15 cmH_2_O leads to a CT recruitment of an average of 10–12% (range 0–40%) depending on the ARDS severity, while in the same condition the recruitment assessed by the gas methods, based on double P/V curves or other gas methods, leads to an overestimation [31]. Regarding the R/I method, recently Murgolo et al. found that it is poorly correlated with absolute recruited tissue (T_rec_), assessed with CT scan, and has no correlation with absolute recruited gas (G_rec_). However, they observed a correlation when the T_rec_ and G_rec_ were normalized to the total lung capacity at the PEEP_low_, as R/I normalizes the compliance of recruited volume to the total compliance at PEEP_low_ [32]. Moreover, Del Sorbo et al. [33], observed a very poor correlation between the R/I and the PEEP-induced decrease in poorly and non-aerated areas.

A key point is understanding the clinical impact of measuring recruitability. Currently, to our knowledge, there are no data comparing the clinical impact of the quantitative CT scan method—which reflects the gas fraction or volume of pulmonary units—with other methods that generally indicate overall lung aeration improvement when a given pressure is applied. The most straightforward application of recruitability assessment is selecting the appropriate PEEP level. However, whether the clinical application of higher PEEP is justified will depend on the interaction between risk/benefits of overinflation/recruitment and hemodynamics (see closing pressure section). What we know in general is that higher recruitability corresponds to more edematous lung (i.e., heavier lung), at least in the early phase of ARDS before structural changes of lung parenchyma may occur.

In this case, the clinical approach should be markedly different, as the potential for lung recruitment is lower, and the effects of PEEP, recruitment maneuvers, and prone positioning may be minimal [34].

### Open lung strategy

The potential for *recruitment* is related to the application of the so-called *open lung strategy*. This in theory suggest that the best treatment to avoid intra-tidal excessive stress and strain, due to the tidal opening-closing pulmonary units, is to keep the lung completely open (i.e., every gasless pulmonary unit must regain inflation). This theory is based on theoretical studies of Mead et al. on stress and strain distribution [35] which was promoted by Lachmann in 1992 [36]. This approach gained a large consensus in the intensive care community because in our opinion it was associated with an immediate effect (the oxygen partial [pO_2_] increasing). However, the stress and strain during open lung strategy were not measured. The effect on gas exchange, specifically improved oxygenation as pO₂ rises, was considered a key indicator of effective recruitment. Consequently, pO₂ was frequently proposed and used as the best marker for selecting PEEP levels to achieve full lung opening. We have concerns in using this approach as the pO_2_ increase may depend simply on the hemodynamic effect as first shown by Danzker et al. in 1980 [37]. Indeed, the best PEEP proposed by Suter et al. [38] was a compromise between respiratory mechanics, hemodynamic and gas exchange. In addition, the inspiratory airway pressure required to fully open the lung, ranges from 45 to 65 cmH_2_O and the PEEP required to keep the lung open is about 25 cmH_2_O. In theory, therefore, we should apply a plateau pressure equal or greater than 45 cmH_2_O and PEEP equal or greater than 25 cmH_2_O. These pressures are intolerable for the hemodynamic point of view.

### Closed lung approach

Within a safe pressure range, part of the lung remains completely closed throughout the respiratory cycle. For instance, if the lung is recruited at 45 cmH₂O and ventilation is maintained with a plateau pressure limit of 30 cmH₂O, any PEEP below 25 cmH₂O would result in the gradual closure of pulmonary units that had opened between 30 and 45 cmH₂O. This means that, despite recruitment maneuvers, some degree of atelectrauma may be unavoidable. It is important to recognise that, even when applying the open lung strategy in ARDS, a portion of the lung will always remain closed during normal ventilation [39]. A strategy using PEEP and tidal volumes that maintain some atelectasis (permissive atelectasis), may protect the lungs (provided they are not subjected to repetitive opening and closing) minimizing hemodynamic compromise and need for fluid resuscitation [39].

## Clinical implications

### Recruitment maneuvers

Often considered for newly admitted patients with worsening respiratory failure, their routine application and clinical efficacy are questionable, and their routine application is not recommended by the ESICM guidelines [40]. Randomized trials indicate poor outcomes when combined with high PEEP [41–43]. This highlights the need to balance the potential of an open lung approach with the difficulty in achieving it without overinflation and the risk of hemodynamic deterioration [44]. In individual cases, or in obesity, the pleural pressure gradient increases due to superimposed pressure, raising the opening/closing pressures and therefore influencing the level of PEEP or inspiratory pressure necessary to achieve or maintain recruitment.

### Sigh ventilation

An alternative is an approach which includes the delivery of one or two breaths with larger volume every minute. This strategy may prevent lung collapse seen with the delivery of low tidal volume ventilation. Sighs maintain lung volumes while allowing PEEP to stay within a reasonable range, around 10 cmH₂O. This has been clearly shown in experimental studies [45] and partially introduced in clinical trial [11]. The sigh ventilation is, in our opinion, a reasonable compromise between hemodynamic, excessive pressure and maintenance of lung volumes.

### Prone position

Prone positioning is sometimes thought to aid recruitment, but it actually redistributes lung pressures, potentially collapsing ventral regions while opening dorsal ones [46, 47]. Its effectiveness as a recruitment maneuver vary by patient and depends on the net balance of dorsal and ventral recruitment. However, it has the effect of homogenizing ventilation and offering additional lung protection. This was demonstrated in patients with moderate to severe ARDS, where prone positioning reduced total lung elastance and enabled comparable EELV and transpulmonary pressures to be achieved at lower airway pressures, thereby reducing mechanical power transfer and improving hemodynamics [48].

## Conclusions

Understanding or estimating recruitability—regardless of the method used, as no single method has shown clinical superiority—provides valuable insight for balancing the reduction of atelectrauma with the prevention of volutrauma.

Different recruitment measurement techniques capture unique aspects of pressure-related lung changes. CT scans using a − 100 HU threshold primarily identify tissues likely to experience cyclic opening and closing, while lowering this threshold to − 500 HU adds complexity without clear benefits. In contrast, respiratory mechanics offer a clearer view of how increased PEEP impacts lung inflation, as they reflect improvements in compliance by both recruiting new lung units and optimising those already open. Recruitment maneuvers can have detrimental effects depending on the balance between achieving recruitment and the risk of hyperinflation, which may lead to extrapulmonary complications mediated by hemodynamic impairment.

Therefore, an individualized global approach to the patient is mandatory: not all recruiters should be recruited.

## Data Availability

No datasets were generated or analysed during the current study.

## Abbreviations

ARDS: Acute respiratory distress syndrome
CT: Computed tomography
LUS: Lung ultrasound sonography
EIT: Electrical impedance tomography
PEEP: Positive end expiratory pressure
ZEEP: Zero end expiratory pressure
P/V: Pressure/volume
EELV: End expiratory lung volume
R/I: Recruitment/inflation
C_rec_: Compliance of the recruited volume
G_rec_: Gas recruited
V_rec_: Volume recruited
T_rec_: Tissue rectruited
pO_2_: Oxygen partial pressure
